# Intraindividual neurophysiological variability in ultra-high-risk for psychosis and schizophrenia patients: single-trial analysis

**DOI:** 10.1038/npjschz.2015.31

**Published:** 2015-09-02

**Authors:** Kyung Soon Shin, June Sic Kim, Sung Nyun Kim, Kyung Sue Hong, Brian F O’Donnell, Chun Kee Chung, Jun Soo Kwon

**Affiliations:** 1 Clinical Cognitive Neuroscience Center, Neuroscience Institute, SNU-MRC, Seoul, Korea; 2 MEG Center, Department of Neurosurgery, Seoul National University College of Medicine, Seoul, Korea; 3 Department of Psychiatry, Seoul National University College of Medicine, Seoul, Korea; 4 Department of Psychiatry, Sungkyunkwan University School of Medicine, Samsung Medical Center, Seoul, Korea; 5 Department of Psychological and Brain Sciences, Indiana University, Bloomington, IN, USA; 6 Department of Brain and Cognitive Science, College of Natural Science, Seoul National University, Seoul, Korea

## Abstract

**Background:**

Intraindividual variability in neurophysiological responses is an important factor in the study of schizophrenia. Interestingly, this variability strongly predicts individual differences in cognitive processing. Neurobiological abnormalities that present during the prodromal phase of schizophrenia are not well characterized. However, these symptoms may provide insight into the key circuits involved in the disorder.

**Aims:**

To investigate the variability in magnetoencephalographic responses at ultrahigh risk and schizophrenia patients.

**Methods:**

Twenty-four ultrahigh risk, 21 patients with schizophrenia and 28 healthy controls were evaluated. The intraindividual variability was estimated by calculating the s.d. of the across-trial amplitude in responses to deviant and standard stimuli. The degree of phase locking across trials was calculated by intertrial coherence.

**Results:**

Greater variability in the responses to deviant and standard tones was noted in the schizophrenia and ultrahigh risk groups compared with controls. Variability in response to standard stimuli was positively correlated with the amplitude for the standard stimuli in all of the groups. Moreover, schizophrenia patients displayed lower alpha and theta intertrial coherence compared with ultrahigh risk and controls. Mismatch negativity amplitude was correlated with the alpha intertrial coherence in all groups. Taken together, the augmented variability and reduced inter-trial coherence provide empirical evidence for increased amplitude and phase inconsistencies in schizophrenia and ultrahigh risk.

**Conclusions:**

The results implicate widespread dysfunction in amplitude modulation and phase concentration in schizophrenia and ultrahigh risk, as well as evidence for early amplitude and phase disruption. These finding suggest intraindividual variability and intertrial coherence appear to be important indicators of pathophysiological processing.

## Introduction

Schizophrenia typically affects individuals during adolescence and early adulthood; however, disturbances in thinking and behavior usually precede the full expression of psychosis. Schizophrenia is known to be preceded by prodromal state, which is characterized by functional deficits and attenuated psychotic symptoms.^1^ For early detection and intervention, ultrahigh risk for psychosis (UHR) concept has been developed during last two decades and studies to prospectively approach the prodromal state.^1,2^ Studies have shown that 15–19% of individuals with UHR state convert to schizophrenia within a 2-year period.^1,2^

UHR individuals demonstrate deficits in attention, executive functioning, working memory, and social functioning. The mismatch negativity (MMN) change is still controversial in UHR subjects. Some studies reported decreased MMN in UHR subjects^3,4^ but others reported intact MMN in UHR subjects.^5–7^ The neural basis for passive auditory oddball paradigm in individuals at UHR, however, is not well understood. Therefore, in addition to find these core deficits, the identification of neurobiological abnormalities in UHR individuals is important.

Increased variability and decreased synchrony in neural circuits are important factors that may contribute to the wide range of cognitive deficits in patients with schizophrenia, and some evidence suggests variability in patients with schizophrenia. Computational evidence has found that patients with schizophrenia may have random spiking that leads to increased neural noise and increased intraindividual variability in postsynaptic potential.^8^ Moreover, behavioral studies have found greater timing variability in schizophrenia patients.^9,10^ Electro- (EEG) and magnetoencephalographic (MEG) evidences have revealed decreased event-related potential (ERP) components amplitude^11^ and ERP topography.^12^ Previous studies also suggested increased variability that may contribute to temporal dysfunction in ERP/MEG components.^13–15^ Some ERP components such as P50 gating, P300 and MMN temporal variability were larger in patients with schizophrenia than in healthy controls.^11,13,16^ These augmented intertrial temporal variability in schizophrenia patients have been related or increased ‘cortical noise’,^11^ which might be considered a function of less arranged neuronal firing.^8^ Schizophrenia is characterized by an abnormally elevated resting state activity in the default mode network.^17^ Moreover, patients with schizophrenia display altered trial-to-trial behavioral variability, physiology, and blood oxygen level-dependent responses in both task related and spontaneous activity. Early researches on increased auditory response variability were revealed in patients with schizophrenia.^18,19^ The altered variability may influence cognitive task performance in schizophrenia patients.^20^ Previous EEG findings have shown greater cortical response variability in schizophrenia compared with healthy controls.^20,21^ In summary, increased variability in behavioral and neural responses may be key features in schizophrenia patients.

MEG and EEG recordings provide noninvasive methods to assess synchrony and oscillatory activity in the frequency domains. MEG activity represents synchronized neural activity across a wide range of frequencies detected on the scalp. Event-related oscillations are analyzed by intertrial coherence (ITC) or phase locking factors. They provide information on MEG signal consistency during events at different frequencies. Previous studies in schizophrenia patients^22^ and UHR subjects have found dysfunctional oscillatory activity.^23^ Therefore, we examined electromagnetic variability and oscillatory activity to facilitate better understanding and identification of prodromal symptoms in schizophrenia before the onset of psychosis.

To our knowledge, previous MEG or EEG studies examining both variability and oscillatory activity elicited by deviant and standard tones have not focused on UHR subjects. The purpose of the present study was to investigate the variability and oscillatory activity during auditory processing of deviant and standard tones in UHR individuals. First, variability was calculated in single-trial MEG in UHR individuals, patients with schizophrenia, and healthy controls. Second, whether coherence or phase locking of oscillatory activity was affected during the UHR state as well as in patients with schizophrenia was examined. We predicted increased intraindividual variability and reduced ITC in patients with schizophrenia, and this could be also found in the UHR subjects as a neurobiological vulnerability marker for their risk for the development of psychosis later.

## Materials and methods

### Subjects

Twenty-four individuals at UHR for psychosis, 21 patients with schizophrenia and 28 healthy controls are summarized in Table 1. Twenty-four UHR individuals were recruited from the Seoul Youth Clinic. The UHR subjects satisfied diagnostic criteria for at least one of three criteria of the comprehensive assessment of at-risk mental states:^24^ (1) attenuated psychotic symptoms (*n*=20); (2) brief limited intermittent psychotic symptoms (none); and (3) genetic risk and deterioration syndrome (*n*=2). Two of UHR subjects had attenuated psychotic symptoms as well as genetic risk and deterioration syndrome. The Structured Clinical Interview for DSM-IV (SCID-IV) Axis I Disorder was administered to identify comorbid psychiatric conditions and to confirm exclusion criteria. To monitor the presence of psychotic features and other symptoms, UHR subjects were also administered the Global Assessment of Functioning (GAF), Positive and Negative Syndrome Scale (PANSS), Hollingshead Scale for parental socioeconomic status, Hamilton Depression Rating Scale, Hamilton Anxiety Rating Scale, and the Family Interview for Genetic Studies. Three UHR subjects were receiving low-dose treatments of atypical antipsychotics, and one was taking antidepressant medication during the baseline assessment; one was taking an anxiolytic drug. Exclusion criteria for all participants included a known history of psychotic illness lasting longer than one week, any lifetime diagnosis of substance abuse or dependence, neurological disease, a history of head injury or medical illness with documented cognitive sequelae, sensory impairments, and estimate full-scale intelligence quotient below 70.

A psychiatrist diagnosed 21 patients with schizophrenia using DSM-IV, the PANSS, and the GAF at admission. Of the 21 patients, 13 were taking antipsychotic medications, 7 were taking both antipsychotics and antidepressants, none was taking an antidepressant alone, and the remaining subject was not taking any medication at the time of the MEG recordings. Only stable outpatients who had not exhibited an increase in symptoms over the past year were recruited.

The healthy controls consisted of 28 subjects who were recruited from an Internet advertisement and via the social networks of hospital staff members and screened using the SCID-IV, non-patient version (SCID-NP), with additional exclusion criterion of any first- or second-degree biological relative with a lifetime history of a psychotic disorder or any serious physical illness, or a first-degree relative with a history of any Axis I disorder.

All of the subjects were instructed to avoid alcohol (for 24 h), nicotine and caffeine (for 4 h) prior to the MEG recordings. The Institutional Review Board at Seoul National University Hospital (SNUH), approved the present study. Written informed consent was obtained from all subjects (or their parents when the subjects were under 18 years old) following a complete description of the intended study. Analysis of MMN for the 18 healthy controls, 16 UHR individuals and 15 patients with schizophrenia has previously been reported.^25^

### Experimental paradigm

Stimuli consisted of 1,200 binaural tones (1,000 Hz, 80 dB, 10-ms rise/fall) administered via binaurally inserted earphones. These tones were differentiated by duration and consisted of infrequent (18.2%, *n*=218) deviant tones (100 ms) and frequent (81.8%, *n*=982) standard tones (50 ms) presented with 300-ms stimulus onset asynchrony. The tones were presented in pseudorandom order using STIM2 software (Neuroscan, El Paso, TX, USA) while subjects were viewed a picture book to divert attention from the auditory stimuli. During the entire MEG session, subjects searched for Wally and Wally’s friends in a picture book called ‘Where’s Wally?’.

### MEG measurements

Continuous MEG was acquired with a 306-channel MEG system (Elekta Neuromag, Oy, Helsinki, Finland) within an electromagnetically shielded room in MEG Center of SNUH. Subjects were seated under the helmet-shaped sensor array. The horizontal and vertical eye movements were observed by electrodes that were placed near the outer canthus and below the left eye. During the MEG recordings, the location of the subject’s head with respect to the sensors was determined by measuring the magnetic field produced by small currents delivered to four head coils. Signals were band-pass filtered between 0.1 and 200 Hz at a sampling frequency of 1001.6 Hz.

### MEG analysis

The temporal signal space separation implemented by Maxfiltersoftware was applied to remove MEG artifacts.^26^ The magnetic fields with 204 gradiometer sensors were separately analyzed off-line for tone stimuli with epochs 100 ms before and 300 ms after stimulus onset. Magnetic counterpart of MMN (MMNm) was obtained by the difference waves between responses to the deviant and the standard stimuli that were derived by averaging only those standards that immediately preceding each deviant. Peak amplitude of MMNm, deviant, and standard responses were averaged across all sensors. Epochs which were contaminated by artifacts with eye blinks and eye movements were rejected by eliminating these sweeps on individual criteria by a visual inspection of each epoch with a range of −100 to 300 ms. At least 160 artifact-free epochs in each condition were averaged. For the sensor level analysis, offline filters applied with a low pass of 40 Hz.

### Intraindividual variability

The intraindividual variability was estimated by calculating the s.d. of the across-trial amplitude under the deviant- and standard-stimulus conditions across the three groups. The intraindividual variability was calculated at each time point in the epoch.

$$\mathrm{Variability}\left(t\right)=\sqrt{\frac{1}{N-1}\sum_{i=1}^{N}{\left(Xi\left(t\right)-\bar{X}\left(t\right)\right)}^{2}}$$

### Intertrial coherence

Temporal spectral analysis of the MEG signal trials was run by applying complex Morlet wavelets.^27^ The width of the wavelet was defined as 7. The wavelet transform was obtained for each individual trial for deviant and standard trials separately, and the absolute values of the resulting transforms from 204 gradiometers for deviant stimuli were averaged. The degree of phase locking across trials was calculated by ITC. ITC was computed to assess the intertrial phase stability for a given time window; frequency bins were used as a measure of neural synchrony during baseline period (−100 to 0 ms) and compared with activity during the post-stimulus period. ITC is a normalized measure describing how the signal phase across trials changes from being uniformly distributed in the range between 0 and 2π (ITC=0) to a phase distribution that is sharply concentrated around the mean (ITC close to 1).^28^ This measure was computed for two distinct frequency bands: theta (5–7 Hz) and alpha (8–12 Hz).

### Statistical analysis

Statistical analyses were conducted using SPSS 19. One-way analysis of variance (ANOVA) was used to test group differences in demographic and clinical variables. An independent *t*-test was used to test for differences between UHR and schizophrenia patients on clinical variables. We compared topographic maps obtained for responses, to deviant and standard stimuli and mismatch fields, among controls, UHR, and schizophrenia patients. For statistical analysis of grand-averaged responses and intraindividual variability, peak amplitude, and peak latency were measured within the interval of 130–240 ms for MMNm, deviant, and standard response for each subjects.

MEG scores were computed for each analytic method (grand-averaged response, variability, and ITC) for each condition (deviant, standard) followed by multivariate analyses of variance (MANOVA) to test the experiment effects. The variables that showed significant main effects were further analyzed by *post hoc* comparisons using Bonferroni Correction. To examine the regional ITC, repeated measures factorial ANOVA was applied, region (left frontal, right frontal, left temporal, right temporal, left parietal, right parietal, left occipital, and right occipital) as within-subject factor, and group as a between-subject factor. The regions were demarcated by sensor location.^23^ *Post hoc* tests were used to determine specific group differences. Pearson correlations were used to investigate relationships among across-trial variability, mean MEG amplitude, ITC values, and clinical measures.

## Results

**Figure 1:**
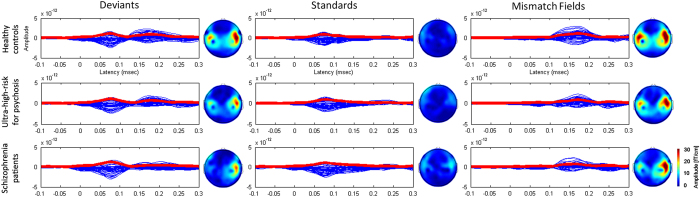
Grand averaged auditory responses were shown as butterfly plots of 204 gradiometers data with epochs 100 ms before and 300 ms after stimulus onset. Topographic maps obtained by grand-averaged responses of deviants, standards and mismatch at 165 ms after stimulus onset.

The grand average responses associated with deviant stimuli, standard stimuli, and mismatch negativity at the sensor level across three groups are shown in Figure 1. For the grand-averaged response analysis, a MANOVA revealed overall effect of group (Wilks’ *λ*=0.028). The main group effects were not significantly observed in MMN (multivariate F(2,70)=2.563, *P*=0.084) and standard (multivariate F(2,70)=1.600, *P*=0.209) responses but deviant condition (multivariate F(2,70)=3.892, *P*=0.025). In *post hoc* analysis of deviant response, there was significant difference between healthy controls and patients with schizophrenia (*P*=0.022).

**Figure 2:**
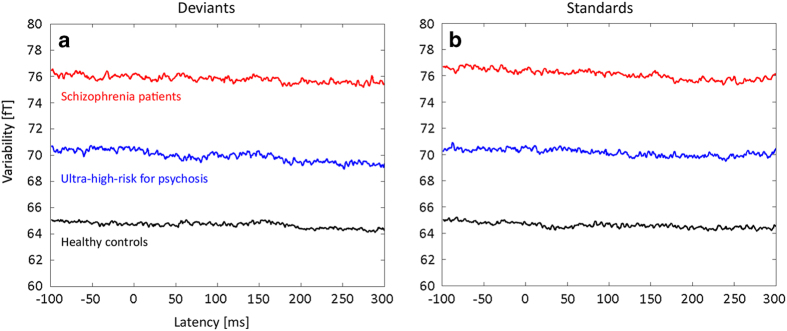
The mean of intraindividual variability is plotted for ultrahigh risk for psychosis (blue), patients with schizophrenia (red), and controls (black) for the (**a**) deviants and (**b**) standards for the time window of −100 to 300 ms.

Figure 2 illustrates the grand mean of intraindividual variability in each group of subjects at each time-point. Patients with schizophrenia showed higher intraindividual variability compared with healthy controls, and the UHR subjects displayed values intermediate between the control subjects and schizophrenia patients under both conditions. In the separate MANOVA analysis on variability in deviant and standard, a MANOVA revealed overall effect of group (Wilks’ *λ*=0.006). Both main group effects of variability in deviant (F(2,72)=6.179, *P*=0.003) and in standard (F(2,72)=5.722, *P*=0.005) were significant. *Post hoc* analysis revealed an increased across-trial variability in schizophrenia patients (*P*=0.001) compared with controls under the deviant condition. *Post hoc* analysis also revealed increased intraindividual variability in schizophrenia patients (*P*=0.002) relative to controls under the standard condition. However, the UHR and schizophrenia and UHR and healthy control groups did not differ from each other under the deviant or standard.

**Figure 3:**
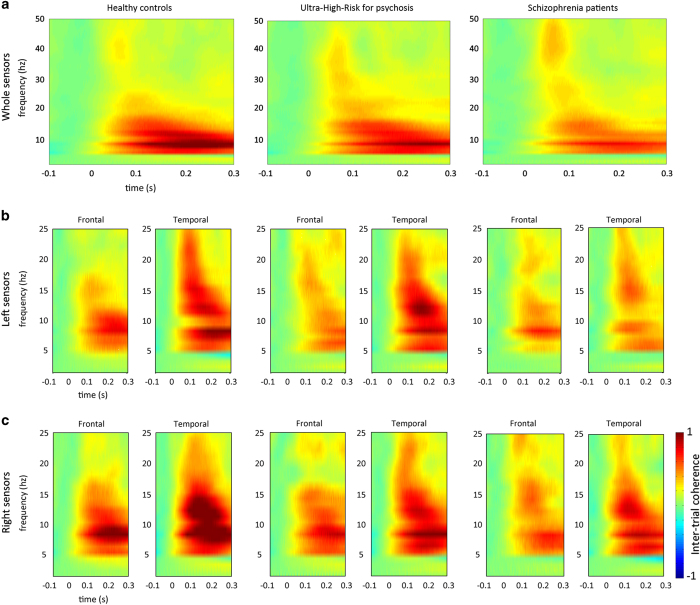
The degree of phase locking on (**a**) whole sensors and (**b**) left and (**c**) right frontal and temporal sensors was calculated by intertrial coherence (ITC) in healthy controls, ultrahigh risk for psychosis, and patients with schizophrenia for the deviant condition.

With related to mean theta and alpha ITC in deviant response, The UHR group displayed values between controls and patients with schizophrenia in deviant responses (Figure 3). We attempted to test mean theta and alpha using a MANOVA in deviant and standard responses. A MANOVA revealed trend toward overall effect of group (Wilks’ *λ*=0.088). The main group effect of mean alpha was significant (F(2,70)=3.932, *P*=0.024) but mean theta was trend level (F(2,70)=3.095, *P*=0.052) in deviant condition. For the standard condition, main group effects were also observed from mean theta (F(2,70)=3.305, *P*=0.043) but mean alpha (F(2,70)=0.289, *P*=0.750).

Repeated measure ANOVAs were conducted for theta ITC across regions in responses from deviants, there was a significant main effect of region (F(7,490)=10.653, *P*<0.001) and a non-significant main effect of group (F(2,70)=2.997, *P*=0.079). No interaction effect between region and group was found (F(14,490)=0.686, *P*=0.789). In responses from standards, there were significant main effects of region (F(7,490)=17.367, *P*<0.001), and trend-significance level group (F(2,70)=2.951, *P*=0.059)). Interaction effect between region and group was not significant (F(14,490)=0.806, *P*=0.663). With regard to the alpha ITC across regions in deviants, we found a significant main effects of group (F(2,70)=3.690, *P*=0.030), and region (F(7,490)=10.346, *P*<0.001), but no interaction effect between region and group (F(14,490)=1.578, *P*=0.081; Figure 3b and c). *Post hoc* analyses revealed that patients with schizophrenia showed reduced alpha ITC compared with healthy control subjects (*P*=0.008). However, no significant differences were detected between the schizophrenia and UHR (*P*=0.109) groups or between the UHR and controls groups (*P*=0.288). Moreover, there were no main effect of group (F(2,70)=0.161, *P*=0.851), and region (F(7,490)=0.659, *P*=0.707) and an interaction between region and group (F(14,490)=0.745; *P*=0.729) for the standard responses.

**Figure 4:**
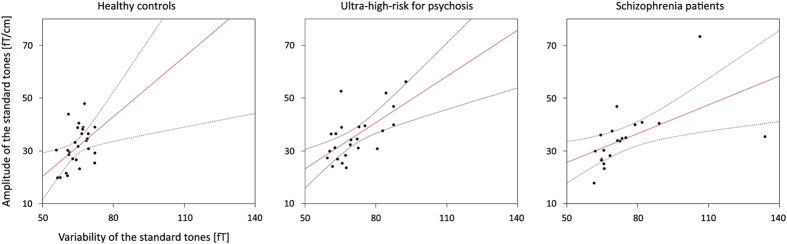
Correlation plots for the variability of the standard condition and amplitude of the standard condition in healthy controls (left), ultrahigh risk for psychosis (middle), and patients with schizophrenia (right). Each dot represents a single subject. Red lines indicate estimation of the best linear fit.

The correlation between the grand-averaged amplitude under each condition and intraindividual variability is shown in Figure 4. A positive correlation was noted between the mean amplitude across trials under the standard condition and intraindividual variability under the standard condition in controls (*r*=0.463, *P*=0.013), UHR (*r*=0.634, *P*=0.001), and schizophrenia patients (*r*=0.561, *P*=0.008). Moreover, a positive association was also detected between mean amplitude across trials under the standard condition and intraindividual variability under the deviant condition in controls (*r*=0.461, *P*=0.014), UHR (*r*=0.624, *P*=0.001), and schizophrenia patients (*r*=0.554, *P*=0.009). The relationship between grand-averaged amplitude and mean ITC was assessed. The MMN amplitude was correlated with the alpha ITC from the left temporal (*r*=0.643, *P*<0.001), frontal (*r*=0.409, *P*=0.030), parietal (*r*=0.493, *P*=0.008) and occipital (*r*=0.427, *P*=0.024) regions in controls. In UHR subjects, the alpha ITC from the left parietal region was correlated with the MMN amplitude (*r*=0.420, *P*=0.041). Correlations between the MMN amplitude and the alpha ITC from the left temporal (r=0.543, *P*=0.011) and frontal (*r*=0.493, *P*=0.003) regions were found in schizophrenia patients. There were no significant correlations among grand-averaged amplitude, variability, ITC, and clinical variables under each condition.

## Discussion

In the present study, we investigated neurophysiologcal variability and oscillatory activity in UHR subjects, patients with schizophrenia and healthy controls using MEG while they performed an auditory passive oddball task. We were particularly interested in analyses of single-trial neural activity. First, we computed variability in single trial MEG in a group of UHR for psychosis, patients with schizophrenia (SPR), and healthy controls (CNT). We confirmed large variability in patients with schizophrenia and UHR subjects compared with controls (CNT<UHR<SPR). Regardless of group, variability in response to standard stimuli was positively correlated with the MEG amplitude in response to the standard stimuli. Second, we analyzed ITC to measure consistency of spectral phase across trials at each frequency and time window. Differences were noted among the three groups in terms of ITC values. *Post hoc* analyses revealed lower alpha and theta ITC in schizophrenia patients compared with UHR and controls. The alpha ITC was correlated with MMN amplitude across regions. Thus, our results indicate increased amplitude and phase inconsistencies in UHR individuals relative to controls. The disruption of neurophysiologcal variability may be important in the expression of a full psychotic syndrome.

Assessment of intraindividual variability using single-trial analysis may provide a window for exploring dynamic modulations in both resting state and cognitive process. Previous electrophysiological findings indicated greater variability in cortical response in patients with schizophrenia compared with healthy controls.^20,21^ Jordanov *et al.* ^13^ explored whether decreased MMN amplitude results from deficient processes or excessive amplitude variability in patients with schizophrenia.^13^ They concluded that increased variability may reflect decreased MMN amplitude in controls but not in schizophrenia patients. As we expected, we observed that schizophrenia patients and UHR subjects showed greater across-trial variability compared with healthy controls. This corresponded to deficiencies in amplitude regardless of group. Our current results on variability expand this finding and suggest that variability may reflect a functional decline in auditory processing among UHR subjects who will later develop psychosis. It is noteworthy that we found a strong correlation between variability under standard conditions and MEG amplitude in response to standard stimuli in every group (Figure 4). In our previous study,^25^ we were able to show the N1m component elicited by standard tones that were presented repetitively. Importantly, the N1m adaptation occurred in healthy controls, whereas neither the UHR nor the schizophrenia group showed adaptation to the repeated stimuli. Since the neurons adapted to the repetitive stimuli, responses to a standard tone were smaller in healthy controls than in patients with schizophrenia or UHR subjects. Consistent with the present findings, enhanced MEG amplitude in response to the standard stimuli was associated with increased intraindividual variability in response to standard stimuli in patients and UHR groups.

The previous study found increased theta and alpha ITC during performance of an auditory change-detection task in healthy controls.^29,30^ The functional role of alpha rhythms has been interpreted as inhibition of task-irrelevant processing.^31^ With regard to the results of the phase alignment, group differences were found in alpha (*P*=0.030) and theta (*P*=0.056) ITC. Several studies have used phase coherence measures and have found a reduction in alpha ITC in schizophrenia patients compared with healthy controls.^32^ Altogether with our previous study showing reduced alpha ITC,^23^ the present findings reflect aberrant ITC of the alpha frequency in schizophrenia and UHR subjects and provide intriguing evidence for disruption of synchronization. These findings suggest that schizophrenia patients and UHR subjects may be characterized by impairments in processing in terms of phase variability in oscillatory activity. These less synchronized responses may contribute to cognitive dysfunction in schizophrenia patients and UHR subjects. Moreover, a positive correlation has been revealed between the enhancement of alpha ITC and MMN amplitude, providing evidence that alpha phase alignment has an influence on the MEG amplitude.

The increased variability and reduced ITC during performance of a passive auditory oddball task, indicates a clear deficit in schizophrenia patients. UHR subjects exhibited mild deficits compared with schizophrenia patients. Taken together, the augmented variability and reduced ITC across single-trials provide empirical evidence of increased variability in amplitude and phase in schizophrenia patients and UHR subjects compared with healthy controls.

The results of this study should be interpreted with caution given several limitations. Some studies reported that reduced MMN observed in UHR subjects^3,4^ indicating that MMN predicts those who will subsequently develop a schizophrenia disorder. However, MMN deficits in UHR subjects not always observed.^5–7^ Discrepancies among studies might be caused by differences in sample characteristics, sample size, and methodology.

Clinical treatments that may influence MMN in schizophrenia and medication use among UHR subjects could not be fully controlled because of the small sample size. Although there is no consistent evidence that antipsychotic medication affects MMN generation, recent study suggests that effects of aripiprazole ameliorate preattentive deficits in schizophrenia.^33^ In the present study, there were three of the UHR subjects (12.5%) who were being treated with atypical antipsychotic medications at the MEG assessment. Although the medication effect for intra individual variability and ITC may be statistically weak, the amount of antipsychotic that the UHR individuals took is not negligible. Future research beyond this limited study is needed to determine whether medication influences these differences in amplitude variability and phase across trials. We did not administer the HAMA scale to two of the schizophrenia patients. Despite this limitation, we still observed significant differences between the UHR and schizophrenia groups.

In summary, with the course of schizophrenia, intraindividual variability and ITC appear to be important indicators of pathophysiological processing. These data indicate widespread dysfunction in amplitude modulation and phase concentration across trials in schizophrenia patients and UHR subjects, and providing evidence for amplitude and phase disruptions.

## Figures and Tables

**Table 1:** Demographic and clinical characteristics

*Variables*	*Healthy controls (*n=*28)*		*Ultrahigh risk (*n=*24)*		*Schizophrenia (*n=*21)*		*Analysis*	*df*
	*Mean*	*s.d.*	*Mean*	*s.d.*	*Mean*	*s.d.*	*F or χ* ^*2*^	
Age (yrs)	23.64	4.09	21.29	3.1	23.67	4.33	3	2
Gender (m/f)	18/10		18/6		16/5		0.58	2
Education (yrs)	14.36	1.47	13.21	1.82	13.48	2.6	2.45	2
IQ	111.36	15.42	108.88	14.5	100.91	9.4	3.70^a^	2
Parental SES	2.89	0.88	3.17	1.17	2.62	0.74	1.87	2
PANSS			58.38	12.48	54.29	12.03	1.24	
CAARMS			40.52	14.4				
GAF	90.93	3.36	53.9	6.96	60.76	11.3	178.92^a^	2
HAMA								
HAMD								
CPZ doses (mg/day)			151.92	215.28	392.21	199.38		
Neuroleptic medication (AP/AD/both/none)				(3/0/1/20)		(13/0/7/1)			

Abbreviations: AD, antidepressant; AP, antipsychotics; CAARMS, comprehensive assessment of at-risk mental states; CPZ, Chlorpromazine equivalent dose; df, degrees of freedom; f, female; GAF, Global Assessment of Functioning; HAM-A, Hamilton Anxiety Rating Scale; HAM-D, Hamilton Depression Rating Scale; IQ, Intelligence Quotient; m, male; PANSS, Positive and Negative Syndrome Scale; SES, Socioeconomc Status; yrs, years.

*χ* ^2^ analysis for categorical data.

^a^ *P*<0.05.
